# Heated tobacco product (IQOS) induced pulmonary infiltrates

**DOI:** 10.1016/j.rmcr.2024.102026

**Published:** 2024-04-27

**Authors:** Merlin Thomas, Mansoor Hameed, Shaikha Alhadad, Irfan Ul Haq

**Affiliations:** a Department of Chest, Hamad General Hospital, Doha, Qatar; b Department of Clinical Medicine, Weill Cornell Medicine -Qatar, Qatar; c Department of Medicine, Hamad General Hospital, Doha, Qatar

**Keywords:** Heated tobacco products, IQOS, Pulmonary infiltrates, Acute lung injury, Eosinophilia

## Abstract

**Background:**

Heated tobacco products (HTPs) have been marketed as safer alternatives to conventional cigarettes, but emerging evidence suggests potential respiratory risks. We present a case of pulmonary complications associated with IQOS, a popular HTP, contributing to the growing understanding of these risks.

**Case description:**

A 40-year-old chronic smoker switched to IQOS, consuming 1.5 packs per day. He presented with incidental chest radiographic abnormalities and peripheral eosinophilia. Computed tomography of chest revealed pulmonary nodules and ground glass opacities. Bronchoscopy indicated mild eosinophilia. After ruling out other causes, a lung biopsy was recommended but declined. Discontinuation of IQOS led to symptom resolution and radiographic improvement. This case adds to a limited literature on HTP-induced lung injury, with a unique presentation and favorable response to cessation.

**Conclusions:**

The case highlights potential pulmonary complications and the first describing an organizing pattern of lung injury associated with IQOS use, emphasizing the importance of recognizing and discontinuing HTPs in patients with respiratory symptoms or radiographic abnormalities. Further research is needed to elucidate the mechanisms underlying the harmful effects of HTPs and inform public health policies. This case underscores the importance of monitoring and educating individuals about the potential risks of HTPs to respiratory health, especially in the context of smokers switching to these products.

## Abbreviations

HTPs: Heated tobacco products
CCs: Conventional cigarettes
AEP: Acute eosinophilic pneumonia
HPHCs: Harmful and potentially harmful compounds

## 1 Introduction

Heated tobacco products (HTPs) are a new class of tobacco products that heat tobacco to a high temperature, but do not burn it. This results in the production of an aerosol that contains nicotine, but not the harmful carcinogens that are produced when tobacco is burned. Hence, HTPs have been marketed as purportedly safer alternatives to conventional cigarettes (CC) [1]. However, emerging evidence suggests that HTPs may still carry potential risks, including several pulmonary complications.

Caputi et al. highlighted an association between HTPs use and respiratory symptoms, including cough and phlegm production [2]. Furthermore, a systematic review by Glantz and Bareham indicated that HTPs aerosols contain a wide range of harmful chemicals, some of which are known to cause lung inflammation and damage [3]. One of the most popular HTPs is IQOS, which is made by Philip Morris International. IQOS is marketed as a "heat-not-burn” tobacco product, and it is sold in over 40 countries [4]. However, there have been several reports of pulmonary problems in people who use IQOS. A recent review described 58 cases of e-cigarette-related respiratory disease, including acute lung injury, organizing pneumonia, eosinophilic pneumonia, and acute respiratory distress syndrome [5]. These studies suggest that HTPs can cause several pulmonary problems.

Despite the limited data on the long-term health effects of HTPs, it is essential to document and analyze specific cases to further our understanding of the potential risks they pose to respiratory health. This case report aims to present and discuss a unique case of pulmonary complications associated with the use of IQOS after switching from CCs, contributing to the growing body of evidence regarding the adverse effects of HTPs on the respiratory system. In the context of HTPs, case reports can contribute to our understanding of the pathophysiology, clinical presentation, diagnostic challenges, and treatment considerations related to pulmonary complications.

## 2 Case description

A 40-year-old Lebanese gentleman was referred from the pre-employment medical commission department for further evaluation of an abnormal chest radiograph. He did not present with any systemic complaints such as fever, cough, chest pain, difficulty breathing, hemoptysis, anorexia, or weight loss. He had no significant past medical, surgical, or family history, except for a sleeve gastrectomy. The patient denied taking any long-term medications, supplements, alcohol, or recreational drugs. However, he had been a chronic smoker for the past 20 years, initially using CCs, smoking a pack per day, but in the last 6 months, he had switched to IQOS and increased his consumption to 1.5 packs of IQOS or more per day over the last few months prior to presentation, due to job-related stress. He recently started working as an entrepreneur and prior to 6 months he was the manager at a printing press with no exposure to printing materials. He does not have any pets at home.

On examination, the patient appeared well built and nourished with vital signs within normal limits. He had no pallor, clubbing, cyanosis, or jaundice on general examination. Chest examination revealed normal bilateral vesicular breath sounds with no added sounds. Other system examinations were unremarkable.

**Fig. 1:**
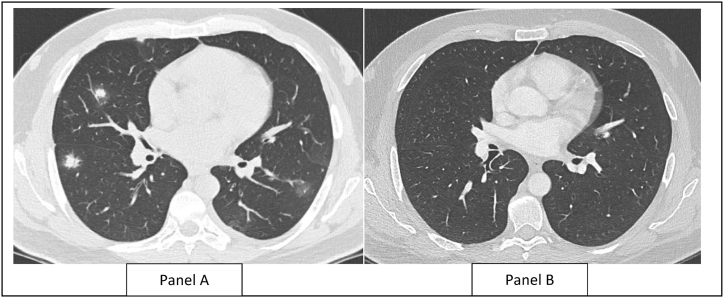
Computed Tomography Chest Images of Pulmonary window at the level of outset of segmental bronchus. Panel A: Variable sized nodular and patchy part solid opacities with surrounding ground glass opacification, showing subpleural and peribronchovascular predominance distribution. Panel B: Resolution of the opacities.

Blood tests revealed leukocytosis, with a white blood cell count of 10.8 × 10^3/μL, and eosinophilia of 3.2 × 10^3/μL. A peripheral smear indicated mild toxic features in the leukocytes, along with some reactive lymphocytes and moderate eosinophilia, possibly reactive in nature. Liver function and renal function tests were within normal limits. Chest radiograph reported ill-defined nodular shadows in the right mid and lower zones. A computed tomography (CT) scan of the chest revealed multiple scattered variable-sized nodules and patchy, partially solid opacities with surrounding ground glass opacification (Fig. 1). These findings were predominantly distributed sub-pleurally and along the peri-bronchovascular region. Centrilobular emphysematous changes were observed bilaterally in the upper lobes, while minimal pleural and pericardial fluid was detected. No significant mediastinal or hilar lymphadenopathy was identified. Bronchoscopy demonstrated normal bronchial anatomy, and the cytology differential showed 89 % macrophages, 5 % neutrophils, 4 % lymphocytes, and 2 % eosinophils. Cultures for bacteria, viruses, fungi, and tuberculosis were negative. Cytopathology results were negative for malignancy.

Considering the differential diagnosis, further assessment with a lung biopsy was recommended, but the patient declined any further invasive interventions. IQOS cessation was strongly advised, leading him to stop using IQOS and switch back to conventional cigarettes. A repeat chest CT scan after 6 weeks showed resolution of the multiple bilateral lung lesions observed in the previous scan. No medications were administered during this period.

## 3 Discussion

In our patient, the presence of peripheral eosinophilia and the observed improvement in nodular pulmonary opacities following cessation of IQOS usage strongly suggests the occurrence of acute lung injury in an organizing pneumonia pattern secondary to IQOS. Through a thorough evaluation, we have systematically ruled out other potential causes, including acute and chronic infections, vasculitis, autoimmune etiologies, or concomitant medications that could lead to lung injury. Notably, our patient remained asymptomatic, and the radiographic findings were incidental. However, given more time, there is a potential for the condition to have evolved into eosinophilic pneumonia, which, to date, is the only pathology attributed to the use of HTPs. The radiological findings our case predominantly correspond to an organizing pneumonia pattern. To our current knowledge, this is the first case report describing and detailing such a pattern of pulmonary injury.

There are only four other documented case reports in the literature describing the development of acute eosinophilic pneumonia (AEP) following exposure to HTPs, as summarized in Table 1 [[6], [7], [8], [9]]. AEP is a rare but serious lung condition that is characterized by inflammation and the accumulation of eosinophils, in the lungs. Most patients with AEP with identifiable causes are current smokers, and changes in smoking habits, such as newly starting smoking, increasing the number of cigarettes smoked, or changing the cigarette brand, can cause AEP [10,11]. AEP potentially induced by switching from CCs smoking to HTPs has only been described in one of the cited cases. All four summarized cases exhibited a relatively short duration of presentation and demonstrated a favorable response to discontinuation of HTPs usage along with a course of corticosteroids. It is noteworthy that one of the reported cases presented as fulminant AEP, necessitating critical care and extracorporeal membrane oxygenation support. The accumulation of these case reports, including our current case, highlights the need for increased awareness regarding the potential pulmonary complications associated with the use of IQOS and similar HTPs. These findings underscore the importance of promptly recognizing and discontinuing HTP usage in patients presenting with respiratory symptoms or radiographic abnormalities. Furthermore, the successful response to corticosteroid therapy in the reported cases suggests a potential role for immunomodulatory treatment in managing such cases of HTP-induced acute lung injury.

**Table 1:** Summary of case reports of HTPs induced lung injury.

Study	Patient	Symptom	Chest Radiograph	CT chest	Bronchoalveolar Lavage	Treatment
Tajiri et al. [6]	47-year-old lady switched from CC to HTP, 4 months prior to referral	Nonproductive cough followed by fever and malaise. Duration: short (exact duration not available)	Bilateral infiltrates	Patchy GGO with interstitial thickening	Eosinophil: 72 %	Prednisolone
Kamada et al. [7]	20-year-old male on HTP for 6 months and doubled the daily use 2 weeks prior to symptoms	Fever and shortness of breath requiring 10L/min oxygen: Duration: 1 day	Bilateral infiltrates	Bilateral infiltration with smooth interlobular septal thickening and pleural effusion	Eosinophil: 60 % Lymphocyte: 20 %	Prednisolone for 2 weeks with dramatic improvement on day 4 radiologically. No recurrence on stopping prednisone after stopping HTP
Aokage et al. [8]	16-year-old man known to have childhood asthma on HTP	Severe cough, fatigue and dyspnea Duration: 2 weeks Progressed to ARDS requiring ECMO despite IV methylprednisolone	GGO on CXR	Consolidation with mosaic pattern	Eosinophil: 14.7 % Neutrophil: 51.7 % Lymphocyte:33.6 %	Methylprednisolone 1 gm IV x 3 days followed by prednisolone 60 mg for 5 days. On ECMO for 10 days with CT chest on day 9 showing healthy lungs
Kang BH et al. [9]	22-year-old lady smoking HTP's for 2 weeks and increased from 6/day to 15/day just before symptoms onset	Duration: 1 day	Bilateral infiltrates	Bilateral multifocal patchy consolidation with smaller nodular GGO and interlobular septal thickening	Eosinophil:62 % Lymphocyte:15 % Macrophage:14 %	Rapid improvement with IV methyl prednisolone with normal CXR at 14 days

HTPs contain tobacco leaves that are heated and therefore have a unique aerosol with distinct toxicity different from e-cigarettes and conventional cigarettes (CC). The product that our patient was smoking, IQOS, was the first HTP launched in 2014. It comprises of disposable heat sticks, which are glycerin-soaked tobacco sticks heated by an electric blade when inserted in a holder [12]. HTPs were created to resemble the taste of CC and serve as an alternative for cases where e-cigarettes failed to create the desired effect of CC authenticity [13]. However, HTPs have been shown in peer-reviewed articles, mostly funded by HTP manufacturers, to reduce exposure to harmful and potentially harmful compounds (HPHCs) that are produced only at combustion temperatures [[14], [15], [16], [17]]. Nevertheless, HTPs contain more than 50 constituents that are cytotoxic and genotoxic, which are not included in the FDA's list of HPHCs, and they are present at significantly higher percentages than in CCs [18].

The portrayal of HTPs as a safe product has created a concerning trend of attracting non-smokers to start using HTPs, in addition to the profile of conventional smokers who want to reduce or stop smoking CC, similar to the profile of electronic cigarettes [[19], [20], [21]]. Because HTPs are less satisfying than CC, they are used more frequently, often exceeding specified doses, although nicotine levels are similar to CC [22,23]. The results of in vitro experiments on human primary keratinocytes and bronchial epithelial cells highlight the comparable cytotoxic effects of cigarette smoke extract from CCs and HTPs, indicating their potential carcinogenicity [24]. HTP aerosol exposure was found to induce cytotoxicity, oxidative stress, and inflammatory responses, with a greater generation of reactive oxygen species observed in intensive HTP exposure scenarios compared to CCs [13]. Transcriptomic changes associated with oxidative stress were more prominent at 4 hours of exposure, suggesting early adaptive mechanisms and antioxidant responses that varied based on the intensity of exposure [13]. Moreover, in a mouse model exposed to an intensive puffing regime of HTPs, characterized by 5 hours of exposure per day over a 2-week period, significant increases in proinflammatory cytokines, chemokines, and CD4+RORγt + T cells were observed in bronchoalveolar lavage fluid [[25], [26], [27], [28], [29]]. These findings shed light on the underlying cellular mechanisms contributing to various inflammatory pathologies like organizing pneumonia in our case, respiratory infections, inflammation, autoimmune diseases, asthma, and chronic obstructive pulmonary disease (COPD).

## 4 Conclusions

The data presented in this study emphasize the potential health risks associated with HTPs, as they induce cytotoxicity, oxidative stress, and inflammation. Further research is warranted to elucidate the specific molecular pathways involved in these processes and to evaluate the long-term effects of HTPs use on human health. Understanding the mechanisms underlying the harmful effects of CCs and HTPs can inform public health policies and interventions aimed at reducing tobacco-related diseases and promoting healthier alternatives.

## Financial support for the case report

10.13039/100019779Qatar National Library funded the article processing charges.

## CRediT authorship contribution statement

**Merlin Thomas:** Conceptualization, Supervision, Visualization, Writing – original draft, Writing – review & editing. **Mansoor Hameed:** Writing – review & editing. **Shaikha Alhadad:** Writing – original draft, Writing – review & editing. **Irfan Ul Haq:** Writing – review & editing.

## Declaration of competing interest

The authors declare that they have no competing interests related to this manuscript. The funding source for article processing charges, Qatar National Library had no involvement in the conceptualization, interpretation, or writing of the manuscript.
